# Large Improvements in the Thermoelectric Properties of SnSe by Fast Cooling

**DOI:** 10.3390/ma18020358

**Published:** 2025-01-14

**Authors:** Andrew Golabek, Nikhil K. Barua, Ehsan Niknam, Luke T. Menezes, Holger Kleinke

**Affiliations:** Department of Chemistry and Waterloo Institute for Nanotechnology, University of Waterloo, Waterloo, ON N2L 3G1, Canada

**Keywords:** chalcogenides, selenides, tin, semiconductors, thermoelectric materials, quenching

## Abstract

As reported during the last five years, SnSe is one of the leading thermoelectric (TE) materials with a very low lattice thermal conductivity. However, its elements are not as heavy as those of classical thermoelectric materials like PbTe or Bi_2_Te_3_. Its outstanding TE properties were revealed after repeated purification steps to minimize the amount of oxygen contamination, followed by spark plasma sintering. Recently, we showed that hot-pressing—once optimized—can yield comparable or even better TE performance using the examples of Na- and Cu- as well as Na- and Ag-co-doped SnSe. However, long-term stability remains a challenge during cycling between low and high temperatures. Here, we investigated whether the cooling procedure has a significant impact on the thermoelectric properties of SnSe. We compared cooling of the melt with a 1:1 ratio of Sn:Se from 1273 K down to room temperature in air with quenching in water. As typical for undoped SnSe, both materials were extrinsic *p*-type semiconductors due to Sn defects. The air-quenched sample exhibited higher thermal conductivity, lower electrical conductivity, and higher Seebeck coefficient, all consistent with a smaller number of defects and thus a smaller number of charge carriers due to the slower cooling procedure. This resulted in a comparatively low peak figure-of-merit value *zT* of 0.61 at 823 K for the air-quenched sample, compared to the substantially higher peak *zT* of 1.58 at 813 K for the water-quenched sample.

## 1. Introduction

The thermoelectric (TE) energy conversion continues to gain more importance for more sustainable energy production [1]. TE materials can generate electricity from waste heat, as used in space applications for several decades [2,3]. This fascinating energy conversion has been investigated for using the waste heat in automobiles to increase gas mileage, as well as in stationary applications [3,4,5,6]. More recently, the researchers’ focus includes powering the billions of sensors in the Internet of Things (IoT) [7,8,9]. The efficiency of the TE conversions depends on the Carnot term and the figure-of-merit, *zT* = *TS*^2^*σ κ*^−1^, with *T* = temperature, *S* = Seebeck coefficient, *σ* = electrical conductivity, and *κ* = thermal conductivity. While classical materials have peak *zT* values around or just above unity [10], larger *zT* values are required for competitive conversion efficiencies. Until recently, most researchers focused on materials composed of heavy elements, such as antimonides and tellurides because of their intrinsically low lattice thermal conductivity in contrast to oxides [11], for example in skutterudites [12,13], Tl_2_Ag_12_Te_7.4_ [14], PbTe [15,16], and Bi_2_Te_3_ [17,18].

Most recently, even bulk SnSe—despite being composed of substantially lighter elements than the above examples—was shown to attain *zT* values above 2.5 after diligent purification steps to remove surface oxides, followed by spark plasma sintering, along with extraordinarily low thermal conductivity at higher temperatures [19,20]. The latter is a result of strongly anharmonic and anisotropic bonding, both a consequence of the lone pair of Sn^2+^ [21]. However, long-term stability may be an issue for SnSe [22,23]. As the processing conditions seem to be of particular importance for SnSe [24,25,26], we investigated with this contribution whether the speed of the cooling from the melt matters for the thermoelectric properties, and checked for stability of the material under the measurement conditions.

## 2. Materials and Methods

To test the effects of different cooling rates, we prepared two samples of undoped SnSe. The starting materials were Sn shots with a nominal 99.6% purity from VWR Chemicals BDH (Mississauga, ON, Canada), and Se shots with 99.999% purity from Central Research Laboratories (South Plainfield, NJ, USA), both stored and handled in an argon-filled glovebox. The Sn shots were first washed for 10 min with 10% HCl prior to use to move surface oxides, dried under vacuum, and then melted in an evacuated fused silica tube at 1273 K. After cooling, the shots were again washed with 10% HCl and then returned into the glovebox, where they were wiped clean.

The quartz tubes were loaded with Sn and Se in the 1:1 ratio and then melted at 1273 K for five hours. The first SnSe tube was air-quenched by removing it from the furnace directly at 1273 K and then placing it onto a firebrick to cool it down fast to room temperature. The second SnSe tube was water-quenched by also removing it from the furnace directly at 1273 K and placing it instantly into a beaker of water to cool down at an even faster rate, i.e., within seconds instead of minutes.

The tubes were returned to the glovebox. Therein, the tubes were opened, and the ingots were ground in an agate mortar until a consistent particle size was achieved. Thereafter, the samples were ball-milled one after the other, under argon for 5 cycles of 5 min at 600 rpm, with ceramic balls in a 1:2 mass ratio, using a Fritsch Pulverisette 7 (Laval Lab Inc., Laval, QC, Canada). Finally, the samples were separately loaded into graphite dies of a diameter of 12.7 mm and hot-pressed for 8 h minutes at 673 K under a pressure of 48 MPa using an Oxy-Gon Industries (Epsom, NH, USA) hot press, followed by a pressure-free cooldown. A flow of a 5% H_2_/Ar mixture was used to remove any O-contamination by reduction during the hot-pressing process.

To check for purity, powder X-ray diffraction (PXRD) patterns were obtained after grinding parts of the hot-pressed samples using the Inel powder X-ray diffractometer equipped with a position-sensitive detector and Cu Kα_1_ radiation.

Energy-dispersive analysis of X-ray (EDAX) measurements was performed using a Quanta FEG ESEM microscope (FEI Company, Hillsboro, OR, USA) with an acceleration voltage of 20 kV. We carried out area scans and elemental mapping on areas of ~300 μm × 300 μm. Several SnSe crystallites were analyzed to check for homogeneity and potential deviations from the nominal 1:1 ratio.

The laser-flash DLF-1200 (TA Instruments, New Castle, DE, USA) was used to measure the thermal diffusivity (*D*) on the pressed disks with a diameter of 12.7 mm and a height of 2 mm under argon. The thermal conductivity was calculated via *κ* = *DρC*_P_, using the Dulong–Petit limit for the specific heat (*C*_P_). The densities (*ρ*) were determined to be above 98% of the theoretical maximum via the Archimedes method. We then calculated the thermal conductivity values, *κ*, via *κ* = *DρC*_P_. Using the ZEM-3 (ULVAC, Methuen, MA, USA), the Seebeck coefficient, and the electrical conductivity were measured under helium on rectangular pellets of 8 × 2 × 2 mm, cut from a second 2 mm disk. In each case, we collected a few additional data points during cooling as well, to investigate the stability, or lack thereof, during the measurement conditions. The errors were estimated to be ±5% for the thermal conductivity [27], ±3% for the Seebeck coefficient, ±5% for the electrical conductivity [28], and therefore, via error propagation, ~10% for the figure-of-merit *zT*: ∂(*zT*)/*zT* = √[2(∂*S*/*S*) + ∂*σ*/*σ* + ∂*κ*/*κ*] = 0.082. The error bars were included in the corresponding figures.

## 3. Results and Discussion

**Figure 1:**
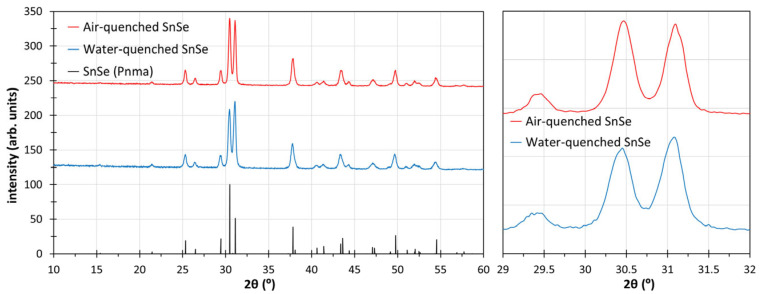
PXRD patterns of air- and water-quenched SnSe. The enlarged region on the right highlights the lack of a shift but a different intensity ratio of the two major peaks above 30°.

SnSe adopts a distorted, layered variant of the NaCl structure, due to the steric effect of the lone pair of Sn^2+^. Above 800 K, the relative orientation of the layers changes, resulting in a symmetry change from space group *Pnma* to *Cmcm* [29]. The PXRD patterns of the air- and the water-quenched samples, obtained after hot-pressing, are compared to each other and to the standard SnSe (low-temperature modification, space group *Pnma*) in Figure 1. No peaks indicative of any side products are visible, and a certain preferred orientation of the crystallites is evident from the intensity changes in the (1 1 1) peak at 2θ = 30.5° and the (4 0 0) peak at 31.1°, compared to the standard pattern. Therefore, there is a noticeable preference for the crystallites to be oriented parallel to the layers in the *b*,*c* plane, which is slightly more pronounced in the water-quenched sample.

**Figure 2:**
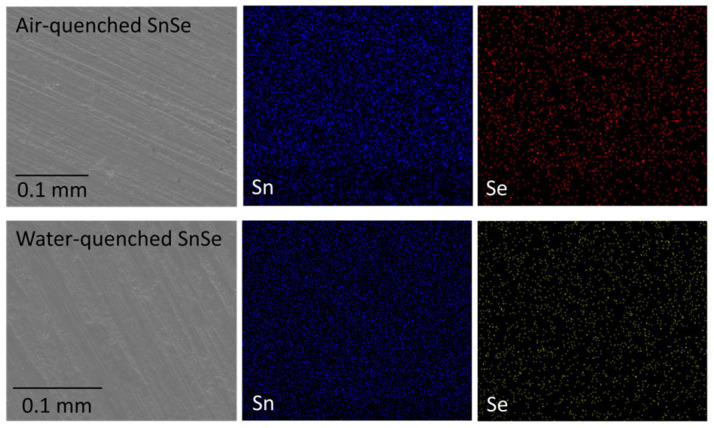
SEM images and elemental distribution of air- and water-quenched SnSe.

EDAX atomic mapping for these materials after the thermal conductivity determination revealed no cracks, and homogeneous distribution of the elements in the expected 1:1 ratio within error based on measurements of 6–8 crystallites, namely 51(2) atomic-% of Sn and 49(2)% Se for the air-cooled sample, and 52(2)% Sn and 48(2) Se for the water-cooled sample (Figure 2).

**Figure 3:**
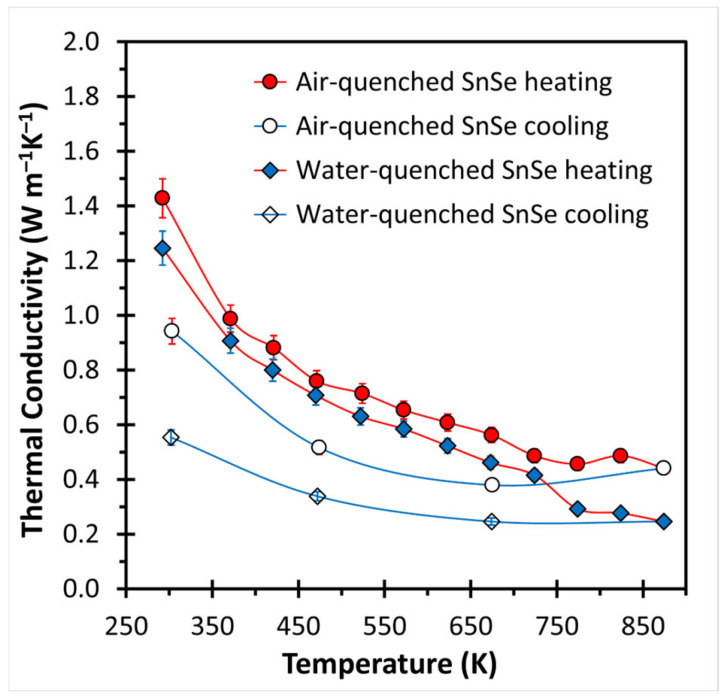
Thermal conductivity of air- and water-quenched SnSe.

The thermal conductivity curves for both heating and cooling—which were obtained parallel to the pressing direction—are shown in Figure 3, noting that we measured beyond the phase transition around 800 K. Both samples exhibit the typical decrease during heating, namely, from *κ* = 1.43 W m^−1^K^−1^ at 293 K to 0.44 W m^−1^K^−1^ at 873 K for the air-quenched sample and from 1.24 W m^−1^K^−1^ to 0.25 W m^−1^K^−1^ for the water-quenched sample in the same temperature range. In both cases, the samples exhibit a hysteresis, where the increase during cooling results in significantly lower values than before, e.g., to 0.94 W m^−1^K^−1^ and 0.55 W m^−1^K^−1^ at room temperature. This is indicative of a lack of stability as observed for other SnSe samples before, which was deduced to stem from crack formation during the heating process [22,23]. Here, the changes are less pronounced, and no cracks were found after the thermal conductivity measurements. The lower *κ* values of the water-quenched sample are likely a consequence of a higher number of defects caused by the faster cooling from the melting. Faster cooling results in less complete ordering and more defects such as Sn atom deficiencies—a known occurrence in SnSe [20]—and site defects such as Sn on Se positions and Se on Sn positions. Both of those defects would increase phonon scattering because of increased mass fluctuations and thereby decrease the thermal conductivity as observed here. Still, all these values are higher than the ones reported for the so far best performing SnSe, namely the double-purified, doped with 2% Na and having a nominal 0.5% Sn deficiency SnSe with *κ* values between 0.6 W m^−1^K^−1^ and 0.2 W m^−1^K^−1^, which reached a peak *zT* value of 3.1 [20]. For comparison, the lattice thermal conductivity of bulk Bi_2_Te_3_ is 1.5 W m^−1^K^−1^, which needs nanostructuring to achieve values comparable with bulk SnSe, such as 0.5 W m^−1^K^−1^ and below [30,31].

**Figure 4:**
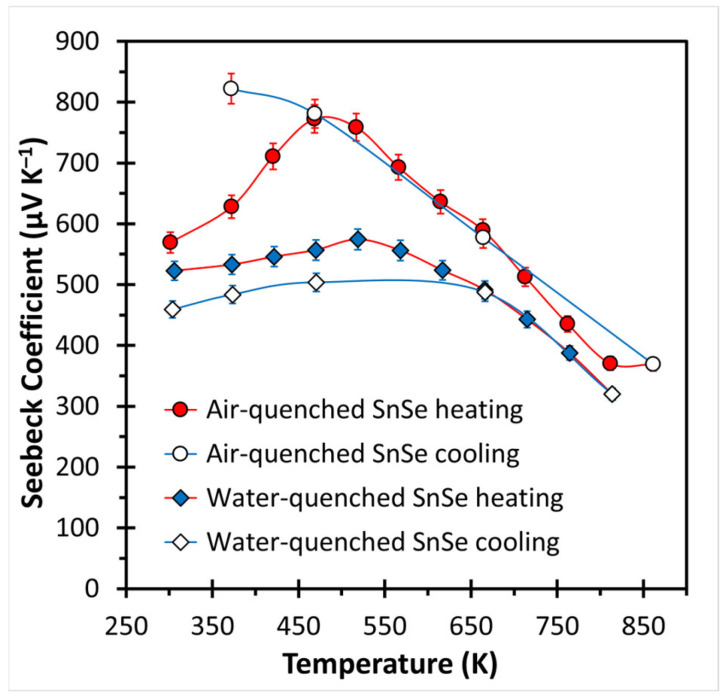
Seebeck coefficient of air- and water-quenched SnSe.

The Seebeck coefficient is positive for both samples (Figure 4), indicative of dominant *p*-type (hole) conduction, typical for undoped SnSe materials because of Sn atom deficiencies [20,22,23]. Both samples exhibit increasing values at first, then decreasing values after 450 K–500 K, which is commonly observed in SnSe materials [20,22,23]. The values, ranging from S = +370 μV K^−1^ to +822 μV K^−1^ for the air-quenched and from +320 μV K^−1^ to +574 μV K^−1^ for the water-quenched sample, are overall higher than for heteroatomic p-doped SnSe materials, such as the above-mentioned, spark plasma sintered 2% Na-doped SnSe with a peak Seebeck coefficient of S = +320 μV K^−1^ [20], or the hot-pressed SnSe co-doped with 1% Na and 1% Cu and the one co-doped with 1% Na and 1% Ag (both with a peak S = +354 μV K^−1^) [23]. A slight but less pronounced hysteresis is observed here as well, notably in the absence of the turnaround point upon cooling for the air-quenched sample, while the turnaround in the water-quenched sample remains noticeable during cooling. That the S values are lower for the water-quenched than for the air-quenched sample is indicative of the above-mentioned larger number of defects (holes) due to the faster cooling process, as each Sn atom deficiency would add *p*-type charge carriers.

**Figure 5:**
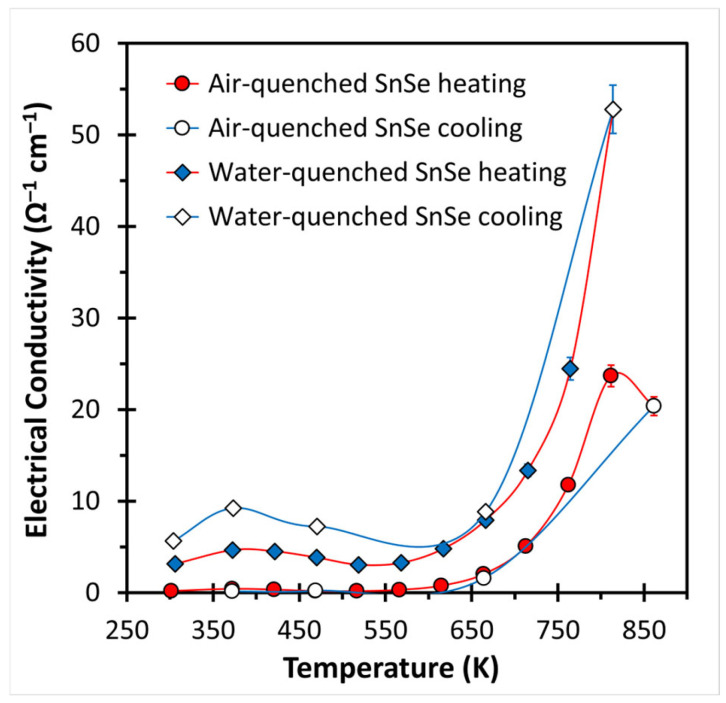
Electrical conductivity of air- and water-quenched SnSe.

The electrical conductivity data, shown in Figure 5, follow the slope often found for SnSe materials, i.e., at first an increase up to about 400 K with local maxima of 0.4 and 4.6 Ω^−1^cm^−1^, then a decrease, followed by a second increase [20,22,23]. Again, the values during the cooling differ notably from the heating process. Just like in the case of the Seebeck values, the turnaround point disappeared in the case of the air-quenched sample, but not in the case of the water-quenched sample. Overall, the water-quenched sample exhibits higher electrical conductivity values, supporting the hypothesis of a higher number of defects (holes). Its values, ranging from σ = 3 Ω^−1^cm^−1^ at 300 K to 53 Ω^−1^cm^−1^ at 813 K, are comparable to the also undoped and purified but spark plasma sintered SnSe published in 2021 with σ ranging from 4 Ω^−1^cm^−1^ to 35 Ω^−1^cm^−1^ (for which a low Hall charge carrier concentration of 2 × 10^17^ cm^−3^ was determined) [20].

**Figure 6:**
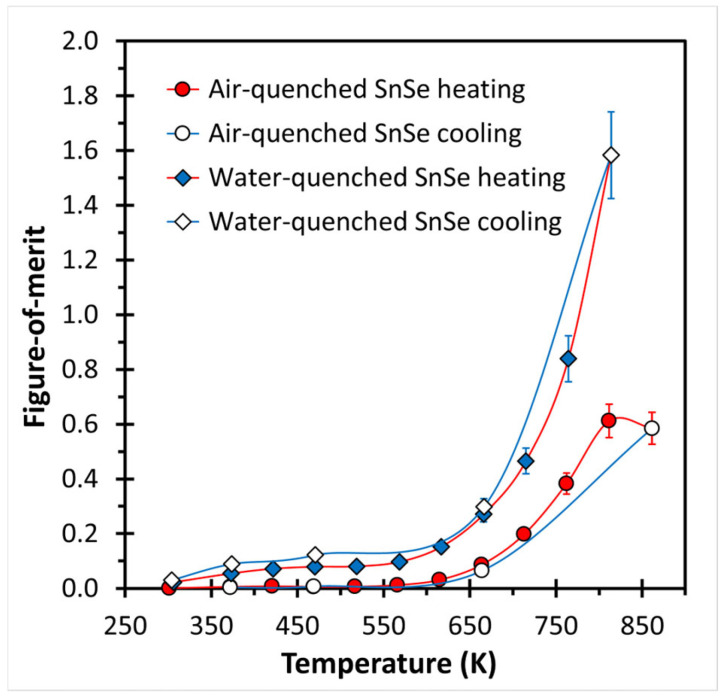
Thermoelectric figure-of-merit of air- and water-quenched SnSe.

Finally, the figure-of-merit is calculated from the Seebeck coefficient, the electrical and the thermal conductivity. First, it should be noted that the *zT* values are restricted to the smaller temperature range of the electrical properties. Second, we note that the samples are anisotropic, the electrical properties were measured perpendicular, and the thermal conductivity was parallel to the pressing direction. On samples with a more pronounced preferred orientation, the electrical conductivity was about 10% lower when measured parallel to the pressing direction, while the Seebeck coefficient remained unaffected [20]. Therefore, the peak *zT* values determined here are overestimated by up to 10%. Third, the slight instability of the samples during the measurements constitutes an additional problem [23]. However, a comparison of these samples with each other, and similarly processed ones, remains instructive. As shown in Figure 6, the *zT* values continue to increase from room temperature up to the phase transition around 800 K, peaking at *zT* = 1.58 at 823 K in the case of the water-cooled sample, and at *zT* = 0.61 at 813 K in the case of the air-cooled sample. Beyond that, the *zT* of the air-cooled sample decreased slightly to 0.58 at 861 K. Even when tentatively adjusting by 10% to correct for the different directions, the peak *zT* value remains outstanding with *zT*_corr_ = 1.58 − 0.158 = 1.42 for the undoped, water-quenched SnSe sample.

## 4. Conclusions

Two different samples of nominally equiatomic SnSe composition were successfully synthesized after purification of Sn by a combination of melting, ball-milling, and hot-pressing, processes that can all be readily scaled up. The different cooling procedures after the melting process turned out to be instrumental in obtaining one sample with high thermoelectric performance. Specifically, cooling in air resulted in a modest peak *zT* value of 0.6, whereas water-quenching yielded a high *zT* value of 1.6 (both above 800 K). The higher performance is a result of the combination of higher electrical and lower thermal conductivity, despite the lower Seebeck coefficient of the water-quenched sample.

As observed in earlier studies, changes in the properties during the cycling of the measurements remain a challenge for potential device fabrication of these materials. However, the stability observed here appears to be higher than in our earlier work with Na-doped SnSe, indicating a dependence on the exact composition of the sample.

## Data Availability

The original contributions presented in this study are included in the article. Further inquiries can be directed to the corresponding author.
